# Integrated Data Analysis Uncovers New COVID-19 Related Genes and Potential Drug Re-Purposing Candidates

**DOI:** 10.3390/ijms24021431

**Published:** 2023-01-11

**Authors:** Alexandros Xenos, Noël Malod-Dognin, Carme Zambrana, Nataša Pržulj

**Affiliations:** 1 Barcelona Supercomputing Center (BSC), 08034 Barcelona, Spain; 2 Department of Computer Science, Universitat Politecnica de Catalunya (UPC), 08034 Barcelona, Spain; 3 Department of Computer Science, University College London, London WC1E 6BT, UK; 4 ICREA, Pg. Lluís Companys 23, 08010 Barcelona, Spain

**Keywords:** data integration, network medicine, network biology, drug re-purposing, matrix factorization

## Abstract

The COVID-19 pandemic is an acute and rapidly evolving global health crisis. To better understand this disease’s molecular basis and design therapeutic strategies, we built upon the recently proposed concept of an integrated cell, iCell, fusing three omics, tissue-specific human molecular interaction networks. We applied this methodology to construct infected and control iCells using gene expression data from patient samples and three cell lines. We found large differences between patient-based and cell line-based iCells (both infected and control), suggesting that cell lines are ill-suited to studying this disease. We compared patient-based infected and control iCells and uncovered genes whose functioning (wiring patterns in iCells) is altered by the disease. We validated in the literature that 18 out of the top 20 of the most rewired genes are indeed COVID-19-related. Since only three of these genes are targets of approved drugs, we applied another data fusion step to predict drugs for re-purposing. We confirmed with molecular docking that the predicted drugs can bind to their predicted targets. Our most interesting prediction is artenimol, an antimalarial agent targeting ZFP62, one of our newly identified COVID-19-related genes. This drug is a derivative of artemisinin drugs that are already under clinical investigation for their potential role in the treatment of COVID-19. Our results demonstrate further applicability of the iCell framework for integrative comparative studies of human diseases.

## 1. Introduction

### 1.1. The COVID-19 Pandemic

The ongoing COVID-19 pandemic caused by a new severe acute respiratory syndrome-related coronavirus (SARS-CoV-2) is an acute and rapidly developing global health crisis that has ravaged many countries worldwide. This virus is highly infectious due to asymptomatic carrier transmission [1,2], and as a result, to date, 620 million people have been infected, and more than six million lives have been lost [3]. Apart from exposing the shortcomings of healthcare systems, this pandemic devastated the economy, and as a result, we are on the verge of a new economic crisis [4,5]. To ease these hazardous consequences, the rapid development of an effective cure that can be applied immediately to reduce mortality or morbidity is needed.

De novo drug discovery, which may last a decade or longer, is not feasible due to the compressed timescale needed for easing the pandemic. Re-purposing the existing drugs is a rapid and effective alternative to provide treatments by re-using drugs that have well-established pharmacological profiles [6]. Hence, from the beginning of the pandemic, the scientific community’s efforts were either to redirect the approved drugs targeting related viruses and assess their efficacy [7] or to develop efficient vaccines. As a result of the global efforts, several vaccines using various technologies have been licensed, and others are under development or clinical trials [8]. At this point, vaccinations have started in the vast majority of the countries, but we are far from the coverage needed to immunize most of the population (i.e., achieve herd immunity). Even if we achieve herd immunity, it is possible that SARS-CoV-2 will become less severe, but more established as a common infection [9]. In addition, the uncertainty about the efficacy of the existing vaccines against the new variants of the SARS-CoV-2 [10,11] makes treatment options a key factor for patients’ health benefits.

SARS-CoV-2 is a (+)RNA virus that depends on the host cells to replicate/propagate by reprogramming the cell to enforce its reproduction [12]. In particular, it reproduces in the upper respiratory tract and it binds to a cellular receptor to enter a host cell, the exopeptidase angiotensin converting enzyme 2 (ACE2) [13]. Upon ACE2 binding, transmembrane protease serine 2 (TMPRSS2) is required to prime the viral spike protein and allow the virus to hijack the host cell via endocytosis [14,15]. The viral proteins interact with 332 host protein targets [16] and so can perform viral functions by modulating cellular processes, such as the regulation of the gene expression and ubiquitination [16]. An inflammatory response to the SARS-CoV-2 infection is evidenced by 1910 differentially expressed host genes (DEGs) in infected lung tissue [17].

### 1.2. Network-Medicine Drug Re-Purposing Methods

From the start of the pandemic, numerous network-based drug re-purposing methods have been proposed (e.g., Zhou et al. [18], Sadegh et al. [19] and Gysi et al. [20] are some of the most cited). In particular, Zhou et al. [18] created an interactome containing drug–target interactions and protein–protein interactions. Since it was a study before the viral–host interactors were published [16], they defined as proteins relevant for COVID-19 those that are direct targets of previous human coronaviruses (HCoV) or are involved in crucial pathways of HCoV infection. Then, they predicted drugs using a proximity measure based on the shortest distance between the drug and the HCoV–host interactions. The approach of Sadegh et al. [19] is based on a group of seed nodes, which can be viral proteins or human genes. It creates a subnetwork containing the seeds and ranks the drugs targeting the seeds using a centrality measure (degree, closeness, betweenness, or TrustRank). Finally, Gysi et al. [20] used the human interactome and prioritized drugs by aggregating the predictions of three different network-based methods: proximity, diffusion and an AI network. These methods have two main limitations: they only predict drugs for genes already known to be related to COVID-19 (or to HCoV in the case of Zhou et al. [18]), and they only use the PPI network as the host molecular network. In a recently published work [21], we used an NMTF-based data-integration framework to bridge the gap between SARS-COV-2 infection mechanisms (the viral–host interactions) and the genes whose expression levels are altered during SARS-CoV-2 infection in humans (the differentially expressed genes in disease, DEGs). To this end, we fused viral–host interactions (for human host), human PPIs, drug–target interactions and drug chemical similarities to identify new drug targets. A limitation of that study was that only data coming from cell lines were fused (viral–host interactions) and that the patient DEGs were only used in the downstream analysis to identify the set of drug targets, rather than in a comparative study of diseased and control tissues.

### 1.3. Comparative Data Integration with iCell

A detailed understanding of the biology of SARS-CoV-2 is required to understand the molecular basis of this disease and to design therapeutic strategies. To understand this basis, we go beyond traditional biological network analysis, and we build upon the recently proposed concept of an integrated cell, iCell [22]. It is based on Non-negative Matrix Tri-Factorization (NMTF) [23] to fuse tissue-specific molecular interaction networks of protein–protein interactions (PPI), gene co-expressions (COEX) and genetic interactions (GI) into an integrated model of a cell. The iCell framework was first applied to construct and compare case (cancer) and control (healthy) tissues for breast, prostate, lung and colorectal tissues to uncover new cancer-related mechanisms or genes.

This comparison revealed genes that were expressed in both cancer and control cells, but whose wirings (i.e., how their patterns of interactions with other genes change) in cancer iCells were altered, whereas they were not necessarily altered in any of the constituent tissue-specific networks. These rewired genes were statistically significantly enriched in cancer drivers. Hence, these wiring alterations in cancer iCells were used to prioritize and predict novel cancer-related genes; among them are genes that could not have been identified using the traditional differential gene expression analysis. The role of these genes in cancer was biologically validated by knockdown experiments followed by cell viability assays. In addition, their role in cancer was also validated in the literature and also through Kaplan–Meier survival curves of thousands of patients.

### 1.4. Contributions

**Figure 1:**
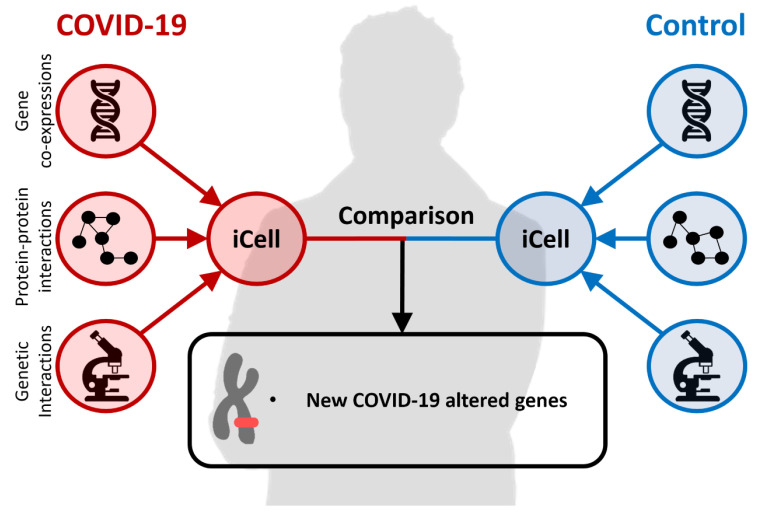
An illustration of the iCell method.

To perform an integrative comparison of patient and cell line responses during COVID-19 infection, we collected the host transcriptional response data to SARS-CoV-2 [17] consisting of expressions in lung samples from COVID-19 positive patients and healthy individuals, and in case and control human cell lines (A549, NHBE and CALU). We applied the aforementioned iCell framework to these data, as illustrated in Figure 1, to create disease and control iCells. The fusion of PPI, COEX and GI networks into an integrated model of a cell differentiates us from the previous studies that used as the host molecular network only the PPI network [18,19,20] and from our previous study [21], in which we created the host molecular network by simply merging (rather than fusing with NMTF) these three different omics data networks. We examined the robustness of our method by analyzing the cell lines and the patient data. We found in patient iCells’ larger discrepancies between control and infected networks than the cell-line-based infected and control iCells, suggesting that the cell lines are not suitable to study the disease and that we should use the data from the human samples instead. Hence, this is what we did in this study and what differentiates us from the already published NMTF-based study [21], also impacting the results.

We demonstrate that iCells, which emerged from the NMTF-based fusion of the molecular networks, better capture the functional organization of infected and control cells than the constituent molecular networks. Comparison between the enriched functions, as captured by Gene Ontology Biological Process (GO-BP) and Reactome Pathway (RP) terms, between the infected patient and control iCells, reveals terms related to the human immune response, such as the cellular defense response and cellular response to interleukin-1, which are only enriched in the infected iCell. Thus, COVID-19 alters the functioning of the iCell by activating its immune response. In addition, it is only the iCell networks, rather than the constituent data networks (PPI, GI and COEX), that are strongly rewired; less than 40% of edges are in common for control and infected networks. The DEGs are statistically significantly more rewired in infected and control iCells than the background genes (the “background genes” are those that are not differentially expressed in COVID-19 infection). Hence, we hypothesize that other intensively rewired genes may also be disease-related, and we prioritized genes according to their rewiring. We validated 18 out of the 20 most rewired genes in patient iCells in “The COVID-19 Drug and Gene Set Library” [24], a database consisting of drug and gene sets related to COVID-19 research. The two newly predicted COVID-related genes, *ZFP62* and *ZNF286A*, both ZINC finger (ZNF) proteins, are likely to be relevant for the disease, since 6 out of the 18 validated genes are also ZNF proteins. In recent studies, it has been shown that the expression of ZNF proteins restricts SARS-CoV-2 infection [25] and that ZNF proteins, as transcription factors, can also activate their target genes to participate in anti-SARS-CoV-2 infection [26]. Interestingly, among the 20 most rewired genes regarding infected iCells, only one gene (*H2AC20*) could have been identified using differential gene expression analysis. In addition, these genes are not highly interconnected in the interactome (PPI network) and thus could not be identified with traditional network-medicine approaches. This demonstrates that our data-integration approach is the only one thus far that could uncover these genes, since it boosts the signal of each of the constituent omics networks. Hence, the main advantage of our method emerges from the data integration (fusion), which provides a more complete view of COVID-19 infection data.

Then, to predict potential candidate drugs to re-purpose for the top 20 newly identified COVID-19-related genes in patients, we go beyond classical drug re-purposing, which is based on using drugs targeting the genes. As demonstrated by Gysi Morelli et al. [20], network-based methodologies are necessary to identify effective drugs that work by perturbing the gene’s subcellular network. Therefore, we applied Graph Regularized NMTF (GNMTF) to fuse the infected-patient iCell with the known drug–target interactions (DTIs) and drug chemical similarities (DCS) to predict potential drugs for re-purposing. We used molecular docking to confirm the ability of the predicted drugs to bind to the predicted targets. Two of the predicted drugs, NADH and fostamatinib, which target our newly identified COVID-19-related genes more often than the other drugs (7 and 5 times, respectively), are already under clinical investigations for their potential roles in the treatment of COVID-19 (as per https://clinicaltrials.gov/ (accessed on 1 July 2022)). For the two newly identified genes, our framework predicts two drugs: artenimol, an anti-malarian drug, a derivative of artemisinin drugs, to potentially target ZFP62, and NADH, to potentially target ZNF286A. Artenimol is an interesting prediction for targeting ZFP62, since this protein is involved in the positive regulation of transcription by RNA polymerase II, which is known to act as an RNA-dependent RNA polymerase (RdRP), and inhibiting RdRP activity is the known mode of action of other widely used COVID-19 drugs, such as remdesivir [27]. Finally, considering the evidence for the predicted drug–protein interaction, ZFP62 and artenimol, and that NADH is already in a clinical trial, we conjecture that our second prediction, ZNF286A targeted by NADH, may also be relevant.

## 2. Results

We created eight condition specific iCells capturing two COVID-19 conditions (infected and control) for one tissue (lung tissue from patients) and three cell lines (A549, NHBE and CALU). To do this, we collected the corresponding gene expression datasets from Blanco-Melo et al. [17], which we used to create condition specific protein–protein interaction (PPI) [28], gene co-expression (COEX) [29] and genetic interaction (GI) [28,30] networks. (see “Creating cell-line and tissue-specific molecular interaction networks” in Materials and Methods). Then, for each condition, we applied the iCell data-integration framework [22] to fuse the corresponding condition-specific molecular networks, yielding eight iCells in total. Note that we excluded from our study the A549-ACE2 cell line, since it is engineered to express the ACE2 receptor and our focus in on the real patient data. The sizes of all the networks are presented in Table 1.

In the following sections, we show that control and infected iCells capture more functional information than the constituent molecular networks (PPI, GI and COEX data), which emerges from the NMTF-based fusion of the networks (detailed in section “COVID-19 and control iCells are biologically coherent” below). Additionally, we show that iCells better capture the rewiring differences between case and control than the constituent data networks (detailed in section “Only iCells are intensely rewired in COVID-19” below). We build upon this observation to prioritize genes according to their rewiring patterns, regarding control and infected iCells, and thus to identify new potentially disease-related genes (detailed in section “Uncovering new COVID-19-related genes with iCells” below). For the 20 most rewired genes in the patient iCells, we predicted drugs for re-purposing by applying the second step of data fusion based on Graph Regularized NMTF (detailed in section “Predicting potential drugs for re-purposing” below).

### 2.1. COVID-19 and Control iCells Are Biologically Coherent

We assessed how well our iCells capture the functional organization of infected and control cells, as described by Gene Ontology Biological Process (GO-BP) [31] and Reactome Pathway (RP) [32] annotations. To do this, for each iCell, we exploited the co-clustering interpretation of NMTF to cluster genes according to the similarity of their wiring patterns in the data (using hard clustering procedure on matrix factor *G*; see details in Materials and Methods, section “Clustering and enrichment analysis”). For comparison purposes, we also applied the same clustering methodology, but when using the iCell framework separately on each constituent data network in isolation from the others; i.e., we obtained clusters of genes that are based on the constituent PPI network, COEX network, GI network or the data fusion of them all (iCell network). In all the cases, the number of clusters, *k*, was set by using the heuristic rule of thumb, $k=\sqrt{\frac{n}{2}}$, where *n* is the number of genes [33]. Then, we measured the enrichments of the obtained clusters of genes in biological annotations (see section “Clustering and Enrichment Analysis”).

**Figure 2:**
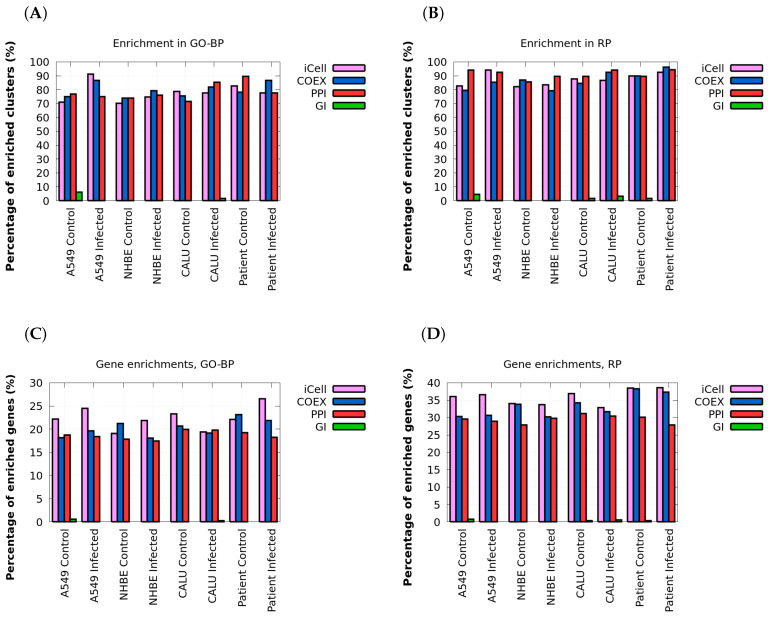
Clusters in iCells have more enriched genes. In panel (**A**), for each tissue and for each network type (x axis) the bar plot shows the percentages of the clusters that have at least one Gene Ontology Biological Process (GO-BP) annotation enriched. Panel (**B**) shows the same, but for Reactome Pathway (RP) annotations. In panel (**C**), for each tissue and for each network type (x-axis), the bar plot shows the percentages of annotated genes in the clusters that have at least one GO-BP annotation enriched in their clusters. Panel (**D**) shows the same, but for RP annotations.

As presented in Figure 2A,B, all networks, except for genetic interaction (GI) networks, have similar percentages of enriched clusters. More than 70% of clusters are enriched in GO-BP annotations, and more than 80% of clusters are enriched in RP annotations. The observed discrepancy between the enrichments obtained when using the GI networks and the ones obtained when using the PPI, and the COEX networks, can be attributed to the smaller number of interactions or sparsity of the GI networks (i.e., the smaller numbers of edges; see Table 1), which makes them less informative compared to PPI and COEX networks. Importantly, iCells are more biologically coherent than any individual molecular network alone, having larger numbers of genes with annotations enriched in their clusters: as illustrated in Figure 2C,D, both infected and control iCells have at least 18% of their genes enriched in GO-BP terms and 35% enriched in RP—more genes than are enriched in the clusters of PPI, GI and COEX networks.

These results confirm the previously observed property in cancer: that iCells capture additional functional information that emerges from the NMTF-based fusion of the molecular networks [22]. However, unlike in the original iCell study, in which cancer networks were less biologically coherent than the control ones in terms of clusters of functionally similar genes [22], the COVID-19 infected networks are not less biologically coherent than the control ones. Importantly, the difference is that although the numbers of enriched GO-BP and RP terms in the infected and control iCells are comparable, the enriched functions are very different. In particular, the Jaccard similarity (a measure of overlap between two word sets) between the enriched GO-BP terms is at most 0.31 and between the RP terms is at most 0.65, for infected and control iCells (see Table 2). In addition, as reported in Supplementary Table S1 in the Supplementary File S1, the enriched functions in the constituent PPI, COEX and GI control and infected data networks are also very different (i.e., the Jaccard similarity between the enriched functions is also small). This demonstrates that COVID-19 alters the functioning of the iCells and their constituent PPI, GI and COEX networks with respect to the control. However, since we have already demonstrated that iCells are more informative and biologically coherent than their constituent networks, we will analyze them further to uncover which functions are altered during COVID-19 infection. The uniquely enriched GO-BP and RP terms for the infected iCells in three cell lines and the patient data are reported in the Supplementary File S2.

As presented in Supplementary Table S2, the number of uniquely enriched GO-BP terms in infected iCells compared to the controls is at least double that of uniquely enriched RP terms in the iCells of cell lines and almost five times larger in the patient iCell. Hence, we focus our analysis on the set of uniquely enriched GO-BP terms to uncover the altered functions in the infected iCells. In particular, we used REVIGO [34] to summarize the list of uniquely enriched GO-BP terms. We observe that terms related to immune response are over-represented in the infected-patient iCell (approximately 25% of the uniquely expressed GO-BP terms). These GO-BP terms include well-known host responses against viral infection, such as positive regulation of natural killer (NK) T cell activation, positive regulation of interleukin-6 production and positive regulation of interferon-alpha production. The over-representation of the immune response processes is in line with the so-called “cytokine storm” produced during the SARS-CoV-2 infection [35]. The hyperactive immune response is characterized by the release of interferons, interleukins, tumor necrosis factors, chemokines and several other mediators [35]. Importantly, the inflammation response produced by the patient is associated with adverse outcomes [36]. Hence, our iCell-based methodology is biologically coherent, and the functional comparison between control and infected iCells confirmed the known mechanisms of COVID-19 infection. In the following sections, we identify which genes drive these functional changes, and we prioritize them as potentially COVID-19-related genes. As a final step, we propose drugs for repurposing targeting their gene products.

### 2.2. Only iCells Are Intensely Rewired in COVID-19

Since the iCells are biologically coherent and capture more information than the constituent data networks, we investigated how their functioning/wiring has been altered in the disease with respect to the control. We demonstrate that only the iCells are intensely rewired in COVID-19 by also investigating the rewiring of each of the constituent PPI, COEX and GI data networks (detailed below).

**Figure 3:**
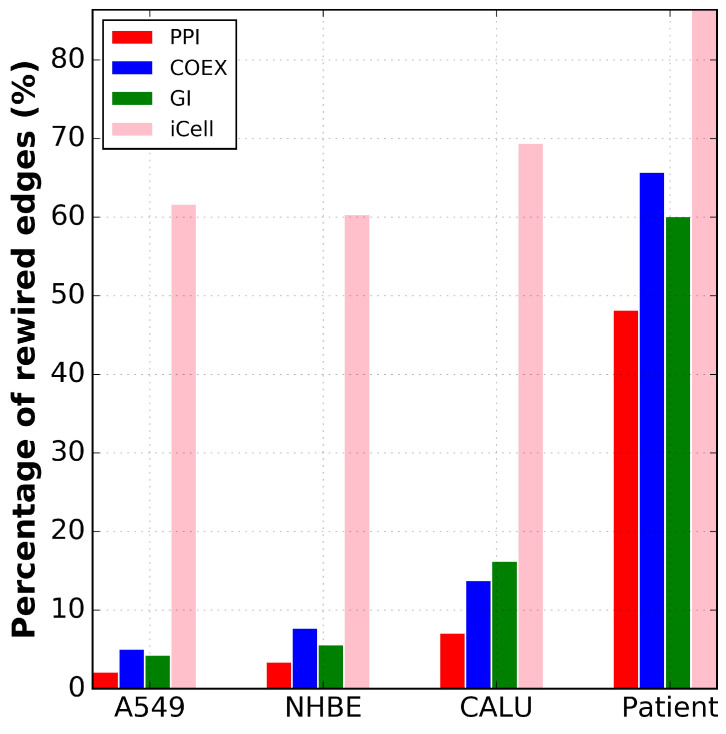
Only iCells are intensely rewired in COVID-19. For each cell line and the patient data (x-axis) and for each type of network (color coded), the percentage of rewired edges (y-axis) between the infected and the control networks is given.

As the first step, we measured the overlap between the networks of control and infected tissues in terms of the overlap between the nodes and edges. As reported in Supplementary Tables S3 and S4 in Supplementary file S1, while the PPI, COEX and GI networks of cell lines are very similar in control and infected conditions (with more than 95% common nodes and 83% common edges), iCells, as presented in Figure 3, are the only networks that are strongly rewired, having less than 40% common edges for control and infected conditions. Thus, cell-line-based iCells better capture the differences between cases and controls than the constituent molecular networks analyzed individually. The patient networks exhibit much larger discrepancies between control and infected networks in terms of common nodes and edges than the networks of cell lines. As shown in Supplementary Table S4 in Supplementary file S1, the percentage of common nodes (genes that are expressed in both infected and control) in the constituent molecular networks and the iCells of patient data is between 51.5% and 57.63%. Interestingly, while the percentage of common edges in the constituent patient data networks varies between 39.9% and 51.77%, it drops to 13.61% in iCells (see Supplementary Table S3 in Supplementary file S1). This suggests that for the patient data, iCells also better capture the differences between cases and controls than any constituent molecular network.

Our results not only show that the functioning of cells is altered by COVID-19 infection, but also indicate that the cell lines may not be suitable to study the disease, because of the large discrepancy between the results for patient and cell-line data. This discrepancy potentially explains why drug re-purposing based on cell lines tends to fail when tested in patients. For example, early in the pandemic, chloroquine and hydroxychloroquine had been used as potential drugs for treatment and prevention of COVID-19 due to adequate results in cell line experiments. However, recent reports [37] from larger trials in patients have shown that hydroxychloroquine did not reduce deaths from COVID-19 and probably does not reduce the number of people needing mechanical ventilation.

### 2.3. Uncovering New COVID-19-Related Genes with iCells

As shown above, the wiring patterns of iCells are different from those of the constituent PPI, COEX and GI networks and are the only ones that are intensely rewired in COVID-19. To assess if the most rewired genes are related to COVID-19, we used graphlets, the most sensitive measure of network topology to date [38], to quantify the rewiring around genes in networks. In particular, we computed the dissimilarity between the corresponding graphlet degree vectors (GDVs) in COVID-19 and control iCells using graphlet degree vector distance (GDVD; see Materials and Methods, section “Capturing the wiring patterns of biological networks”). We examined if the genes whose protein products bind to the SARS-Cov-2 proteins [16] (termed viral–host interactors (VHIs)), and the differentially expressed genes (DEGs) in COVID-19 [17] are significantly more rewired than the other genes (termed “background genes”) between infected and control iCells. To do so, we compared the distribution of the GDVDs of the VHIs and of the DEGs with the distribution of the GDVDs of the background genes. These distributions are statistically significantly different if the *p*-value of the non-parametric Mann–Whitney test is smaller than or equal to 0.05.

As presented in Table 3, in iCells of all examined cell lines and patient data, the VHIs were less rewired than the background genes (with *p*-value $\leq 3.96\times 10^{-3}$) in all iCells except for CALU-cell-line iCell (*p*-value ≈ 0.15). This shows that VHI genes do not directly alter the functioning of the cell. On the other hand, DEGs were significantly more rewired than the background genes (with *p*-value $\leq 4.03\times 10^{-3}$), which suggests that not only was the expression of these genes altered during the infection, but also their functioning, since their interacting partners changed. We built upon this observation by hypothesizing that other intensely rewired genes in iCells may also be COVID-19-related, so we prioritized genes according to the extent of their rewiring.

For each cell line and the patient data, we prioritized the 100 most rewired genes in the iCells of control versus infected cells. Then, we computed the pairwise overlap between the 100 most rewired genes among all the studied samples. As shown in Supplementary Table S5 in Supplementary S1, the overlap between the 100 most rewired genes in iCells of the cell lines is, on average, 18.3 genes. However, the overlap between the rewired genes in patient data and cell lines is at most 11 genes. This overlap is statistically significantly smaller than the aforementioned overlap in the cell lines (*p*-value =0.032; we used the two-sample Kolmogorov–Smirnov test to compare the distributions of the overlapping genes). This discrepancy further confirms that the cell lines are unsuitable for studying the disease. Thus, we focus only on the most rewired genes in the patient data.

In the previous iCell-based study [22], the most rewired genes were not differentially expressed—i.e., their role in the disease was not connected to changes in the transcription. To assess whether our 100 newly prioritized genes are differentially expressed, we first found how many of them are DEGs based on the data from the lung samples of COVID-19-infected patients [17]. Among the 100 most rewired genes, only five (ANXA3, HIST2H2AC, IRF2, LBH and TNNC1) were DEGs. To further examine this, we also collected transcriptomic data (RNA-Seq) from blood samples of COVID-19 infected patients and healthy individuals from the study of McClain et al. [39] (GEO accession number, GSE161731). From the bulk RNA-Seq data, we computed the DEGs using the *limma* package [40] (for details, see “Differentially expressed genes from RNA-Seq data” in Materials and Methods) and identified five other genes (LIPT1, PFDN5, RBAK, TFDP1 and ZNF302) as DEGs. Thus, only 10 of our 100 newly prioritized genes were differentially expressed. Hence, our iCell-based analysis was complementary to the differential expression analysis and identified genes whose transcriptional patterns have not changed due to COVID-19 but that are important for the disease.

Having verified that our method identifies genes that are not differentially expressed but are related to the COVID-19 infection, we explored if these genes could have been identified with traditional network medicine approaches. These approaches are based on the assumption that the cellular components associated with a disease aggregate in the same neighborhood of the PPI network (i.e., in the human interactome), forming disease modules (clusters) [41]. However, in the control and the infected PPI networks, the 100 most rewired genes are not inter-connected; only six of them are direct neighbors. Moreover, their average distance is 3.96 in the control PPI network and 4.12 in the infected PPI network. These distances are higher than the average shortest path distance in both PPI networks, which is 2.84. Finally, these genes are not central in the PPI network: their average betweenness centrality is $4.78\times 10^{-5}$, which is smaller than the average betweenness centrality in the network ($1.93\times 10^{-4}$). Hence, these newly identified genes are scattered in the PPI networks, not forming modules (clusters), and hence could not have been identified with traditional network-based approaches that rely on clustering (modularity) in a single type of omic network.

Then, we performed literature curation for the 20 most rewired genes in patient iCells. We validated 18 of these genes in “The COVID-19 Drug and Gene Set Library” [24], a collection of drug and gene sets related to COVID-19 research (see Table 4, column External Validation). The high validation rates confirm that our methodology is able to uncover COVID-19-related genes from patient-tissue-based experiments. Importantly, 6 out of the 18 validated genes code for zinc finger (ZFN) proteins (e.g., ZFN35, ZFN41, ZNF189 and ZNF597). ZFN proteins are involved in a wide range of molecular functions, such as transcriptional regulation, ubiquitin-mediated protein degradation, signal transduction, DNA repair, cell migration and the immune response [42]. Thus, SARS-CoV-2 infection may activate the response of the human cells by these proteins. This indicates that the two new genes (also ZNF proteins) that have never been associated with COVID-19, ZFP62 (a zinc finger protein involved in nucleic acid binding) and ZNF286A (another zinc finger protein that has DNA-binding transcription factor activity), are likely to be relevant for the disease. In addition, recent studies have shown that expression of ZNF proteins restricts SARS-CoV-2 infection [25] and that ZNF proteins, as transcription factorsm can also activate their target genes to participate in the host response against SARS-CoV-2 infection [26].

Furthermore, the majority of the ZNF proteins reported in Table 4 participate in the herpes simplex virus (HSV-1) infection pathway. This observation could indicate that either the human cell responds similarly to both viruses, or that HSV-1 is reactivated in SARS-CoV-2 patients. The detection of pulmonary HSV-1 in the later phase of SARS-CoV-2 infection has been widely reported. It occurs in parallel with increases in CD38+, HLADR+ and CD8 T-cells and decreased expression of interferon-stimulated genes [43]; in other words, with dysregulation of the patient’s immune response produced by the primary infection. Although whether this reactivation has an impact on disease severity remains an open question, recent studies hypothesized that without the help of HSV-1, the SARS-CoV-2 virus may not be able to cause serious illness or death in humans [44]. Thus, prophylactic treatment to contain HSV-1 [44], or the control of the ZNF proteins [25], could be vital in the fight against SARS-CoV-2.

Apart from the ZNF genes, we also found other interesting therapeutic targets among our most rewired genes. We found that the pathways affected by the dysregulation of the 20 most rewired genes in patient iCells correlate with the majority of the confirmed COVID-19 clinical symptoms, such as anosmia [46] (OLFM2 and REEP4), myopathy [47] (KLHL9), renal deficiency [48] (NUP85) and changes in the immune response [35] (CBX5 and CSTF2T). Other genes are related to symptoms that are still under investigation, such as neurological sequels [49] (CYB561 and LCMT2) and infertility [50] (RPSAP58 and ASRGL1).

Our results demonstrate that the changes in the wiring patterns of the molecular networks during COVID-19 infection, as captured by graphlets in iCells, can uncover new disease-related genes. These genes are mainly related to the host’s immune response to infection. Importantly, these newly identified genes could not have been identified with either network-medicine- or differential-expression-based approaches that rely on a single type of omic data. This demonstrate the power of our data fusion approach and its ability to identify disease-related genes. A key remaining question is the potential usage of these genes as drug targets for the treatment of COVID-19, which we investigated next.

### 2.4. Predicting Potential Drugs for Re-Purposing

To predict potential candidate drugs to re-purpose for our 20 prioritized genes (COVID-19-related), we applied the second step of data fusion based on Graph-regularized Non-negative Matrix Tri-Factorization (GNMTF). It is inspired by a similar framework that was successfully utilized for ovarian cancer to stratify patients, predict novel cancer-related genes and propose drugs for re-purposing [51]; and more recently also for COVID-19, to understand SARS-CoV-2 infection mechanisms and to propose drugs for re-purposing [21]. However, in that study, we only fused data from cell lines with drug–target interactions. In addition, the patient DEGs were only used to identify the set of the common neighbor genes rather than to create disease and control tissues. In the approach presented in this paper, to overcome these limitations, we first generated disease and control iCells. Then, we used graph-regularized NMTF to fuse patient iCells with the data on known drug–target interactions (DTIs) and drug chemical similarities (DCS). We used this framework to predict drugs for re-purposing that target the most rewired genes between the patient control and disease iCells. The COVID-19 patient iCell and the DTI network are represented by their adjacency matrices, $G_{inf}$ and $R_{12}$, respectively; the DCS network is represented by its Laplacian matrix, *L*. The DTI matrix is decomposed into low-dimensional matrix factors $R_{12}\approx G_{inf}H_{12}G_{2}^{⊤}$, where $G_{2}$ is the drug matrix factor (for details, see Equation (1)). The network structure (topology) information from the DCS network was incorporated into the data fusion by using the regularization term, $tr(G_{2}^{⊤}LG_{2})$ (see Section “Predicting New Drug-Target Interactions”).

Before using our framework to predict novel DTIs for COVID-19, first we validated that it captures the relationships between the drugs, i.e., that the drug cluster indicator matrix, $G_{2}$, groups together drugs having similar DrugBank “Drug Category” (DC) annotations. We did this by following the approach of Zambrana et al. [21]; we clustered the drugs by applying the hard clustering procedure to the drug cluster indicator matrix, $G_{2}$, and then we measured the enrichments of the produced clusters in DC annotations (for more details, see Materials and Methods, section “Clustering and enrichment analysis”). As illustrated in Supplementary Figure S1 in Supplementary File S1, more than 75% the drug clusters are enriched in drug categories, for both the cell lines and the patient data, indicating that our approach produces functionally coherent results. To assess if the observed enrichment is greater than or equal to an enrichment that may be obtained by chance, we performed a permutation test (detailed in Materials and Methods, section “Clustering and enrichment analysis”). The enrichments of the drug clusters are statistically significantly larger compared to those in the randomly generated clusters (100 permutations, *p*-value ≤0.01), confirming that our clustering of drugs is meaningful. Thus, we exploited the drug clusters to predict novel drug–target relations.

In particular, we used this framework to predict drugs for re-purposing, targeting the 20 most rewired genes in patient iCells. To predict new, previously unobserved drug–target interactions, we used the matrix-completion property of the reconstructed drug–target relation matrix, $\hat{R_{12}}\approx G_{inf}H_{12}G_{2}^{⊤}$. Each entry of the reconstructed matrix (i.e., a drug–target pair) contains an association score, *s*, which can be interpreted as a relative measure of confidence for each drug–target association. We consider a new entry (that is, not in the original DTI matrix) as a predicted DTI if its association score is higher than the mean of the scores of the already existing DTIs. Based on that criterion, the predictions for the top 20 rewired genes are shown in Table 4 (column, Potential drug for re-purposing). The most frequent drugs in the list are NADH, a nutraceutical targeting eight genes, and fostamatinib, a drug initially used to treat chronic immune thrombocytopenia, targeting five genes. NADH is the reduced form of NAD+, and both are the forms of the coenzyme nicotinamide adenine dinucleotide (NAD), which is involved in numerous energy metabolism pathways, such as glycolisis [52]. NAD+ levels decline with aging, which might be a major contributor to the age-dependent severity of COVID-19 symptoms [53]. In a recent study [54], it was suggested that a deficiency of NAD+ may be a primary factor related to the SARS-CoV-2 disease spectrum that increases the risk for mortality. NAD+ deficiency impairs SIRT1 function, modulating cytokine production; the uncontrolled production of cytokine, the so-called cytokine storm, has been related to severe symptoms of COVID-19 [55]. Thus, nutritional support with NAD+ and SIRT1 activators could minimize disease severity if administered prophylactically or therapeutically [54,56]. The other most frequently predicted drug, fostamatinib, is already under clinical trials for its potential role as a treatment against COVID-19 (in 3 studies at https://clinicaltrials.gov/ (accessed on 1 July 2022)).Thus, our most frequently predicted drugs are already being investigated in clinical trials or in other studies for their roles against COVID-19.

For the two newly predicted COVID-19-related genes, ZFP62 and ZNF286A, there do not exist any drugs targeting their gene products. Hence, we applied our data integration framework and predicted artenimol, an anti-malarian drug, as potentially targeting ZFP62, and NADH as potentially targeting ZNF286A. The interesting one is artenimol, a derivative of artemisinin [57] drugs. This family of drugs is already under clinical investigation for their potential role in the treatment of COVID-19. Artenimol targets ZFP62, a zinc finger protein involved in the positive regulation of transcription by RNA polymerase II, which is known to act as an RNA-dependent RNA polymerase (RdRP) [58]. Importantly, inhibiting RdRP activity is the known mode of action of other COVID-19 tested drugs, such as remdesivir [59], and of other drugs recently proposed for repurposing for COVID-19, such as suramin [60]. Thus, there is evidence that both the drug (artenimol) and the target (ZFP62) are relevant to the disease, further indicating the relevance of our predicted drug–target interaction of artenimol with ZPF62. Finally, the relevance of ZFP62–artenimol and the ongoing research on NADH’s role in treating severe COVID-19 symptoms implies that the other predicted drug–target interaction, ZNF286A targeted by NADH, may also be relevant for COVID-19.

We further validated our 20 highest-scoring drug repurposing predictions using molecular docking (we used the state-of-the-art docking method AutoDock Vina v1.2 [45] with its default parameters, as detailed in Materials and Methods, section “Predicting new drug–target interactions”). Note that we could not find a experimentally validated, or predicted protein structure for the RPSAP58 gene, so we could not perform the docking for it. Additionally, we excluded from the drugs the small chemical compounds (zinc chloride, n-formylmethionine and acetylsalicylic acid). As presented in Table 4 (column, Binding free energy), the binding free energy values for all the predicted DTIs are all strongly negative, ranging from −7.7 to −13 kcal/mol. To interpret these results, we converted the free energy to the dissociation constant, $K_{d}$, which relates to the drug concentration. The lower the $K_{d}$ value (lower concentration), the higher the binding affinity of the drug. As illustrated in Supplementary Table S6 (column, Dissociation constant), all our proposed DTIs have favorable binding activities, including fourteen drug repurposing predictions with strong binding affinities in the nanomolar range ($10^{-7}\leq K_{d}\leq 10^{-9})$ and two predictions with moderate binding affinities in the micromolar range ($10^{-4}\leq K_{d}\leq 10^{-6})$. All of our predicted DTIs have a very small dissociation constant, $K_{d}$, which confirms that the drugs can bind to the predicted targets. These favorable docking results further confirm the ability of predicted drugs to bind to the predicted targets.

For the completeness of the study, we provide in Supplementary File S3 the list of the drugs for repurposing targeting the gene products of the 100 most rewired genes. The most frequently recommended drugs for repurposing are fostamatinib, targeting 22 gene products; NADH, targeting 20; zinc, targeting 16; and rtrenimol, targeting 15. Apart from the already discussed drugs (fostamatinib, NADH and artenimol), zinc is a supplement that can reduce mortality in patients with severe pneumonia [61] and has been already under clinical trials for its potential role as a treatment against COVID-19 (in 77 studies at https://clinicaltrials.gov/ (accessed on 1 July 2022)). Among this extended list of predicted DTIs, we distinguish HIST1H1C, a histone-related gene, targeted by artenimol, since this gene is related to the transcriptomic immune profile of COVID-19 patients [62]. In addition, we found that the gene products of 73 out of the 100 most rewired genes are targeted by drugs already under investigation for a role in the treatment of COVID-19. These high literature validation rates indicate that the remaining DTIs for the 100 most rewired genes in COVID-19 (in Supplementary File S3) are also disease-related, which we provide for future validations to the scientific community.

In conclusion, our methodology can uncover new potential disease genes and also predict potential drugs targeting their protein products, opening the way to new treatments.

## 3. Discussion

We show that the iCell methodology is not only applicable to studying cancer [22] but also to studying COVID-19, uncovering new disease-related genes that could not have been identified by traditional differential expression analysis. In particular, among the top 100 prioritized genes, only ten could have been identified using differential gene expression analysis (we used blood and lung samples). This verifies that our approach is complementary to the traditional differential gene expression analysis in the context of human diseases. When we analyzed the protein–protein interactions of the top 100 prioritized genes, we observed that the newly identified disease-related genes are not highly interconnected and do not form a disease module in the PPI network (basic assumptions of network medicine). Hence, these genes could not be identified when each data type is considered in isolation, further demonstrating the power of data fusion.

Our data-integration framework is versatile and can be extended to include more types of omics data. For instance, if there were available time-series data for the disease, we could have created time-resolved control and infected iCells. By comparing these time-resolved iCells, we could uncover the altered genes at each stage of the infection and identify the disease’s potential drivers. By comparing different time-resolved iCells, it would be easier to separate disease-induced from disease-causing genes, which are not always separated when comparing the transcriptome of diseased and healthy subjects [63].

Furthermore, we extended the iCell methodology to predict, for the newly identified disease genes, potential drugs for re-purposing to target their protein products. Interestingly, none of our top 20 prioritized genes and none of the seven drugs that we predicted to target them were identified in our previous integrative study of COVID-19 [21]. This highlights the complementarity of the two approaches; despite the fact that they both use NMTF to integrate biological data, they are two different heuristics that gain new and different insights into COVID-19.

We believe that our findings pave the way for new treatments that may be necessary due to the unknown efficacy of the existing vaccines against the new variants of the disease [10,11]. However, a limitation of this study is that the newly proposed COVID-19-related genes and the predicted drugs for re-purposing need to further be validated by wet-lab experiments. Finally, our extended iCell methodology is universal and could be used to analyze any disease that has molecular data available that are similar to the data used in this study.

## 4. Materials and Methods

### 4.1. Creating Cell-Line and Tissue-Specific Molecular Interaction Networks

We collected three human molecular interaction datasets: experimentally validated protein–protein interactions (PPIs) from BioGRID version 3.5.182 [28], genetic interactions (GIs) from BioGRID version 3.5.182 [28] and SynLethDB [30], and gene co-expressions (COEXs) from COXPRESdb version 7.3 (file name: *Hsa-m.c7-0*, the one containing the higher number of samples). We also collected diseased and control tissue-specific gene expression data from Blanco Melo et al. [17]. In that study, we only considered genes whose expression value was measured and that have at least one reported protein–protein interaction in BioGRID (as PPIs are the most direct evidence that genes interact).

For each cell line and for each molecular interaction dataset, we generated a tissue-specific molecular interaction network in which nodes represent genes (or their protein products) that are expressed in the cell line, and in which nodes are connected by edges if the corresponding genes interact in the corresponding molecular interaction dataset. Note that we considered a gene to be expressed in a given cell line if its expression value, $log_{2}\left(TPM+1\right)$, is greater than or equal to 1 in 50% or more samples. In this way, we obtained three (PPI, GI and COEX) cell-line-specific molecular interaction networks for each tissue.

We used the same procedure to create tissue-specific networks of lung samples from COVID-19 positive patients and for SARS-CoV-2 infected cell lines: A549, NHBE and CALU. We also created the corresponding control networks (not infected) in the same way. The sizes of the generated networks are presented in Table 1.

### 4.2. Gene Annotations

From the Reactome database [32], we collected the Reactome Pathway (RP) annotations of the human genes. We also collected from Gene Ontology [31] the Biological Process (GO-BP) annotations of the genes. All annotations were collected in March 2020. In addition, from BioGRID database [28], we collected the list of 332 proteins that are the interactors of SARS-CoV-2 proteins [16]. Finally, we collected the list of 1910 differentially expressed genes (DEGs) in the lungs of COVID-19 infected patients [17].

### 4.3. Differentially Expressed Genes from RNA-Seq Data

We collected expression data (RNA-Seq) from blood samples of COVID-19 infected patients and healthy subjects from the study of McClain et al. [39] (GEO omnibus: GSE161731). We used the *limma* package [40] to compute the differentially expressed genes. To pre-process the RNA-Seq data, we used the raw read counts in counts per million (CPM) and filtered out the little-expressed samples and genes. Using log-CPM, we computed the Q1 median of all samples and used it as a threshold to remove those samples with their Q3 lower than or equal to this threshold. After filtering, we normalized the RNA-Seq data using the methods suggested by Ritchie et al. [40]: trimmed mean of M-values [64] and variance modeling at the observational level [65]. Then, to compute the DEGs, we used the procedure of *limma*, a gene-wise linear model, meaning that each gene was tested independently to check whether the expression data of the infected samples was up or down-regulated with respect to the healthy samples.

### 4.4. Drug Data

We collected the drug-related data from the DrugBank database (version 5.1.3) [66]. We obtained 3895 drug–target interactions (DTIs) between the $n_{1}=5916$ gene products (proteins) in our infected-patient iCell and the $n_{2}=8279$ drugs (FDA-approved and experimental). These interactions were captured by the DTI relation matrix $R_{12}^{n_{1}\times n_{2}}$. We also collected the Simplified Molecular-Input Line-Entry System (SMILES) information of these $n_{2}$ drugs to create the drug chemical similarity (DCS) network. Namely, we used the Tanimoto similarity coefficient [67] to compute the pairwise chemical similarity between the SMILE representations of the drugs. Then, we created the DCS network by retaining only the top 5% the most similar drug pairs, which resulted in 1,727,436 links.

### 4.5. Creating Cell-Line and Tissue-Specific iCells

All molecular interaction networks, *i* (PPI, GI and COEX), are represented by their adjacency matrices, $A_{i}$, symmetric matrices in which entry $A_{i}\left[u\right]\left[v\right]$ equals one if genes *u* and *v* interact in network *i* and equals zero otherwise. Following iCell’s data-fusion framework [22], all adjacency matrices, $A_{i}$, were simultaneously decomposed into products of three matrix factors, G, $S_{i}$ and $G^{T}$, as: $A_{i}\approx G\cdot S_{i}\cdot G^{T}$, where *G* is interpreted as the cluster indicator matrix of genes (grouping *n* genes into *k* clusters) that is shared across all decompositions and hence allows learning from all data, and $S_{i}$ was interpreted as the compressed representation of network *i* (that indicates how the *k* clusters of genes relate to each other in network *i*). In this study, we set the number of clusters, *k*, using the rule of thumb $k=\sqrt{\left(n/2\right)}$, where n is the number of genes [33].

This decomposition was performed by minimizing the following Multiple Symmetric Non-negative Matrix Tri-Factorization (MSNMTF) objective function:

$$\min_{\left(S,G\geq 0\right)}\sum_{i}∥A_{i}-G\cdot S_{i}\cdot G^{T}{∥}_{F}^{2},$$

where $∥\cdot {∥}_{F}$ denotes the Frobenius norm.

This minimization problem is computationally intractable (as a polynomial of order 6), and thus we heuristically solved it with a fixed point method that, starting from an initial solution, iteratively uses multiplicative update rules to converge towards a locally optimal solution [22].

After minimization, following iCell’s methodology solution [22], we used the obtained matrix factors to create an integrated network that encompasses all input networks. This integrated network was obtained by thresholding the matrix $G\cdot G^{T}$ by using row- and column-centric rules to preserve only the strongest 1% of relationships in each row and column. In the co-clustering interpretation of NMTF, each row of *G* corresponds to a gene, each column of *G* corresponds to a cluster and the value G[u][i] (in row *u*, column *i*) is the closeness of gene *u* to cluster *i*. We extracted clusters of genes from *G* by using the hard clustering procedure [68], in which gene *u* is assigned to the cluster *C(u)* to which it is closest in *G*, i.e., $C\left(u\right)={argmax}_{i=1}^{k}G\left[u\right]\left[i\right]$.

#### 4.5.1. Clustering and Enrichment Analysis

In the co-clustering interpretation of NMTF, each row of matrix factor *G* corresponds to a gene, each column of *G* corresponds to a cluster and the value G[u][i] (in row *u*, column *i*) is the closeness of gene *u* to cluster *i*. We extracted clusters of genes from *G* by using the hard clustering procedure [68], in which gene *u* is assigned to the cluster *C(u)*, to which it is closest in *G*—i.e., $C\left(u\right)={argmax}_{i=1}^{k}G\left[u\right]\left[i\right]$.

We assessed the biological relevance of the clusters of genes produced by the iCell framework by using the following enrichment analysis. For a given iCell, we extracted the clusters of genes from matrix factor *G* by using the hard clustering procedure described above, and we measured the percentages of these clusters that are enriched in Gene Ontology Biological Process (GO-BP) or Reactome Pathway (RP) annotations.

The probability that an annotation is enriched in a cluster is:

p=1−∑i=0X−1KiM−KN−i/MN,

where *N* is the size of the cluster (only annotated genes from the cluster are taken into account), *X* is the number of genes in the cluster that are annotated with the annotation in question, *M* is the number of annotated genes in the network and *K* is the number of genes in the network that are annotated with the annotation in question. A cluster is significantly enriched if the enrichment *p*-value, after Benjamini–Hochberg correction for multiple hypothesis testing, is lower than or equal to 0.05. We also measured the quality of the clustering by computing the percentage of genes having at least one of their annotations enriched in their clusters of all annotated genes.

To assess if an observed enrichment is greater than or equal to enrichment by chance, we randomly shuffled (permutated) the values in the gene matrix factors. Then, we computed the times, *r*, that a permutation (i.e., a random enrichment) has enrichment greater than or equal to the observed one. We repeated this process for n=100 times and we computed the p-value of the permutation test as $p=\frac{r+1}{n+1}$. We considered an enrichment to be statistically significant if the corresponding p-value was lower than or equal to 0.05.

In the Results section, “Predicting potential drugs for re-purposing,” we followed the same procedure to obtain clusters of drugs from matrix factor $G_{2}$ and to measure the percentages of the produced clusters that are enriched in Drug Categories (DC) (collected from DrugBank [66]).

#### 4.5.2. Capturing the Wiring Patterns of Biological Networks

As graphlets are the most sensitive measure of network topology to date [38], we used them to capture the local wiring patterns around nodes in networks. Graphlets are small, non-isomorphic, induced sub-graphs of a large network that appear at any frequency [69]. Within a graphlet, symmetrical groups of nodes, called automorphism orbits, are used to characterize different topological positions that a node can participate in. These orbits are used to generalize the notion of node degree: the graphlet degrees of a node are the numbers of times a node is found at orbit positions [70]. Following the methodology of Yaveroglu et al. (2014) [71], we used the 11 non-redundant orbits of 2- to 4-node graphlets, which have been shown to perform better than when including higher order graphlets. Thus, each node in a network was characterized by an 11-dimensional vector called the graphlet degree vector (GDV), which captures the 11 non-redundant 2- to 4-node graphlet degrees of the node.

Within a network, we quantified the similarity between the wiring patterns of two nodes by using the graphlet degree vector distance (GDVD) [72] between their GDVs, which we computed as follows. Given two GDV vectors, *u* and *v*, the distance between their *i*th coordinates is defined as:

$$D_{i}\left(u,v\right)=w_{i}\times \frac{|\log\left(u_{i}+1\right)-\log\left(v_{i}+1\right)|}{\log(\max u_{i},v_{i}+2)},$$

where $w_{i}$ is the weight of orbit i that accounts for dependencies between orbits [72]. Then, GDVD is defined as:

$$GDVD\left(u,v\right)=\frac{\sum_{i=1}^{11}D_{i}\left(u,v\right)}{\sum_{i=1}^{11}w_{i}}.$$

GDVD is a distance in [0,1), such that a distance equal to 0 means that the two GDVs are identical.

#### 4.5.3. Predicting New Drug-Target Interactions

To predict potential drugs for re-purposing, for the most rewired genes in the patient iCell, we used a data fusion framework that is based on Graph-regularized Non-negative Matrix Tri-Factorization (GNMTF) method [51]. By using this method, we fused the matrix factor $G_{inf}$ corresponding to the COVID-19 infected case with drug-target interactions (DTIs) and Drug Chemichal Similarity (DCS) data obtained from DrugBank. The DCS network is represented by its Laplacian matrix, $L^{n_{2}\times n_{2}}$, computed as: L=D−A, where *A* is the adjacency matrix and *D* is the diagonal degree matrix of the infected iCell matrix, $G_{inf}$. Namely, we decomposed the DTI relational matrix, $R_{12}$, into a product of three non-negative low-dimensional matrices, $G_{inf}$, $H_{12}$ and $G_{2}$, where $G_{2}$ is the drug matrix factor and $H_{12}$ is a compressed representation of the network $R_{12}$. During the decomposition, we took into account the known structure of the DCS network by adding a regularization term, $tr(G_{2}^{⊤}LG_{2})$, so that $G_{2}$ favors grouping together drugs that are chemically similar.

These low-dimensional matrices can be obtained by solving the following optimization problem:

(1) $$\min_{G_{2}\geq 0}J=\min_{G_{2}\geq 0}\left(∥R_{12}-G_{inf}H_{12}G_{2}^{⊤}{∥}_{F}^{2}+tr\left(G_{2}^{⊤}LG_{2}\right)\right),$$

where $∥\cdot {∥}_{F}$ denotes the Frobenius norm and *tr* denotes the trace of a matrix. To minimize the objective function, *J*, we used a fixed point method that initializes the matrix factors $G_{inf}$, $H_{12}$ and $G_{2}$, with the singular value decomposition (SVD) based strategy [73] and iteratively uses the multiplicative update rules to converge towards a locally optimal solution.

To predict new drug–target interactions, we exploited the matrix completion property of the GNMTF framework. Namely, after obtaining the low-dimensional matrices, the reconstructed drug–target interaction matrix, $\hat{R_{12}}=G_{inf}H_{12}G_{2}^{⊤}$, is more complete than the initial matrix $R_{12}$. Each entry in the reconstructed DTI matrix, $\hat{R_{12}}$, can be interpreted as an association score, *s*, for each drug-gene pair—the higher the score, the stronger the association. We consider a new entry (i.e., an entry that is not in the original DTI matrix, $R_{12}$) as a predicted DTI if its association score is higher than the mean of the scores of the already existing DTIs. For the genes that do not have a predicted DTI based on this selection strategy, we report the drug with the highest association score.

To assess if the predicted drugs can bind to the protein targets, we performed molecular docking using AutoDock Vina [45]. For each drug, we collected its 2D chemical structure from the PubChem database [74], and for each protein (gene product), we collected its experimentally-determined 3D structure from RCSB Protein Data Bank (RCSB PDB) [75]. For the proteins that did not have experimentally validated structures, we collected their predicted ones from the AlphaFold Protein Structure Database [76]. Following the AutoDock Vina documentation, we removed the water molecules from the experimental protein structures from PDB and added the hydrogen bonds. Then we imported both the drug and the protein into the AutoDock Vina and performed the docking using the default parameters.

## 5. Conclusions

To sum up, we applied a versatile data integration framework to the host transcriptional response data to SARS-CoV-2 [17], from patient and cell lines data, to construct the COVID-19-infected iCells and their corresponding controls. We observe that patient iCells exhibit larger discrepancies between control and infected networks than the cell-line-based infected and control iCells, suggesting that patient iCells are more suitable for studying the disease. We demonstrate that iCells not only capture the functional organization of infected and control cases, as measured by clustering and enrichment analysis, but also capture additional functional information that emerges from the NMTF-based fusion of several different types of molecular data. In addition, we show that iCells are the only intensely rewired networks with less than 40% common edges between control and infected iCell networks; i.e., iCells better highlight the differences between cases and controls than their constituent omics data networks in isolation. By comparing the enriched Gene Ontology Biological Process (GO-BP) terms in the infected and control patient iCells, we confirmed that COVID-19 alters the functioning of the infected iCell with respect to the control by activating the immune response.

We built upon these observations and compared the infected and control iCells to identify the most rewired genes in COVID-19. We demonstrated that the DEGs are the most intensely rewired genes, and hence, we prioritized genes to be COVID-19-related according to their extent of rewiring between these iCells. We validated 18 out of the top 20 the most rewired genes in patient iCells in “The COVID-19 Drug and Gene Set Library.” We applied the second step of data fusion to predict drugs for re-purposing for our newly identified COVID-19-related genes, which we validated with molecular docking. The most frequently predicted drugs were NADH, targeting eight genes, and fostamatinib, targeting five genes; both are already investigated for their roles against COVID-19. An interesting predicted DTI is artenimol, an antimalarial agent targeting ZFP62, one of our newly identified COVID-19-related genes. This drug is an interesting prediction resulting from our analysis, since it is a derivative of artemisinin drugs that are already under clinical investigation for their potential roles in the treatment of COVID-19, hence validating our approach.

## Figures and Tables

**Table 1:** Sizes of the networks. For each network, the table shows its numbers of nodes (column #Node) and edges (column #Edge).

	PPI		COEX		GI		iCell	
	**#Node**	**#Edge**	**#Node**	**#Edge**	**#Node**	**#Edge**	**#Node**	**#Edge**
Infected A549	9623	178,828	9286	593,544	6968	22,418	9623	837,077
Control A549	9592	177,728	9253	591,607	6970	22,903	9592	829,609
Infected NHBE	9391	174,892	9074	565,177	6933	22,027	9391	788,520
Control NHBE	9531	177,648	9204	585,361	7095	22,863	9531	822,374
Infected CALU	9434	175,830	9301	599,549	6957	20,505	9434	805,284
Control CALU	9149	169,229	9021	564,297	6536	18,391	9149	745,167
Infected Patient	5916	90,631	5845	241,213	3743	8978	5916	319,549
Control Patient	9552	168,284	9420	609,304	6739	20,143	9552	806,876

**Table 2:** Enriched functions in infected and control iCells are different. For each cell line/patient (column 1), we computed the number of enriched functions (GO-BP and RP, column 2) in the control (column 3) and the infected (column 4) cells, and the Jaccard similarity (column 5) between the enriched functions in the control and the infected iCells.

Cell Line	Annotation Type	#Enriched in Control	#Enriched in Infected	Jaccard Similarity
	GO-BP	1193	1520	0.31
A549	RP	832	872	0.65
	GO-BP	1201	1046	0.28
NHBE	RP	752	852	0.57
	GO-BP	1028	929	0.24
CALU	RP	743	723	0.65
	GO-BP	1145	828	0.21
Patient	RP	862	618	0.53

**Table 3:** Only DEGs were significantly rewired between control and infected iCells. For each cell line and the patient data (column 1), we computed the average rewiring of VHIs (column 2), DEGs (column 3) and background genes (column 4). We compare the rewiring of the VHIs and the DEGs with the background genes, and the entries are statistically significant are in bold (the corresponding *p*-value of the Mann–Whitney test is in parentheses).

Cell Line	Rewirement of VHIs	Rewirement of DEGs	Rewirement of Background Genes
A549	0.037	**0.064** (*p* value <0.01)	0.044
NHBE	0.038	**0.063** (*p* value <0.01)	0.043
CALU	0.053	**0.072** (*p* value <0.01)	0.057
Patient	0.073	**0.088** (*p* value <0.01)	0.085

**Table 4:** Twenty most rewired genes in patient iCells. For each of the 20 most rewired genes in patient iCells of infected versus control (column 1), we report the number of other studies that have reported it as a COVID-19-related gene (column 2) and if it is a differentially expressed gene (column 3). In addition, we report if it is already targeted by an FDA-approved drug (column 4) and the potential drug for re-purposing based on our framework (column 5). Finally, for each predicted DTI, we report its binding free energy (column 6) computed using AutoDock Vina v1.2 [45]. Note that we could not find a experimentally validated or predicted protein structure for *RPSAP58*, so we could not perform the docking for it. Additionally, we excluded from the drugs the small chemical compounds (zinc chloride, n-formylmethionine and acetylsalicylic acid).

Gene	External Validation (#Studies)	Diff. Exp.	Existing Drug (Drugbank)	Potential Drug for Re-Purposing	Binding Free Energy (kcal/mol)
ZNF35	8	No		NADH	−9.8
RPSAP58	3	No		NADH	-
ZNF562	1	No		NADH	−9.4
OLFM2	5	No		FOSTAMATINIB	−9.6
CYB561	8	No		ZINC CHLORIDE	-
ZNF41	4	No		FOSTAMATINIB	−8.5
LCMT2	5	No	LEUCINE	N-FORMYLME THIONINE	-
CSTF2T	3	No		NADH	−10.8
NUP85	11	No		CLADRIBINE	−7.2
REEP4	9	No		FOSTAMATINIB	−9.3
ASRGL1	6	No	ASPARTIC ACID ASPARTACIAL	NADH	-9.7
ZFP62	-	No		ARTENIMOL	−7.6
CBX5	10	No	COPPER	ACETYLSALICYLIC ACID	-
KLHL9	7	No		ARTENIMOL	−10.6
ZNF189	6	No		FOSTAMATINIB	−9.9
ZNF597	4	No		NADH	−10.8
H2AC20	7	Yes		ARTENIMOL	−8.2
CSTF1	1	No		FOSTAMATINIB	−13
ZNF507	9	No		NADH	−8.6
ZNF286A	-	No		NADH	−10.7

## Data Availability

The data and algorithms presented in this study are available online at https://gitlab.bsc.es/axenos/covid-icell/, accessed on 1 November 2022.

## Supplementary Materials

The following supporting information can be downloaded at: https://www.mdpi.com/article/10.3390/ijms24021431/s1.

## Abbreviations

The following abbreviations are used in this manuscript:

SARS-CoV-2	severe acute respiratory syndrome-related coronavirus
PPI	Protein–protein interactions
COEX	Gene Co-Expressions
GI	Genetic Interactions
GO	Gene Ontology
DC	Drug Category
VHI	Viral–host interactions
DTI	Drug–target interactions
DCS	Drug–chemical similarity
SMILES	Simplified Molecular-Input Line-Entry System
DEGs	Differentially expressed genes
GO-BP	Gene Ontology Biological Process
RP	Reactome Pathways
NMTF	Non-Negative Matrix Tri-Factorization
MSNTF	Multiple Symmetric Non-negative Matrix Tri-Factorization
GNMTF	Graph Regularized Non-Negative Matrix Tri-Factorization
iCell	Integrated cell
SVD	Singular value decomposition
