# Supplementary material for: Integrated Data Analysis Uncovers New COVID-19 Related Genes and Potential Drug Re-Purposing Candidates
Source: Int J Mol Sci. 2023 Jan 11;24(2):1431. doi: 10.3390/ijms24021431 (PMC9863794; doi:10.3390/ijms24021431)
Supplement: Supplementary file 1 [file ijms-24-01431-s001.zip › S1 File-MDPI_Special_Issue_Supplementary.pdf]

Supplementary Material for Integrated data analysis uncovers new COVID-19 related genes and potential drug re-purposing candidates

Alexandros Xenos[1,2,†], Noël Malod-Dognin[1,3,†], Carme Zambrana[1,2]
Nataša Pržulj[1,3,4*]

[1]Barcelona Supercomputing Center (BSC), 08034 Barcelona, Spain.

[2]Universitat Politecnica de Catalunya (UPC), 08034 Barcelona, Spain

[3]Department of Computer Science, University College London, WC1E 6BT London, United Kingdom.

[4]ICREA, Pg. Lluís Companys 23, 08010 Barcelona, Spain.

This Supplementary Material document contains Supplementary Figure S1 and Supplementary Tables S1 to S5.

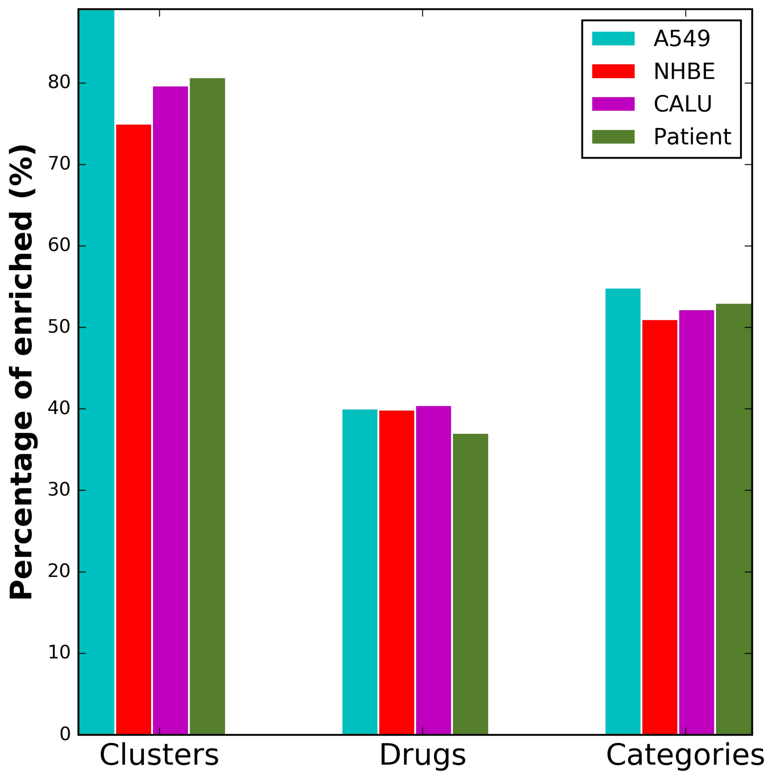

Supplementary Figure S1: **Drug matrix factor enrichments.** For the patient and for each cell-line (color coded), we compute three types of enrichments: the percentage of clusters of drugs having at least one of the Drug Categories (annotations) of the drugs in the cluster enriched (denoted by "Clusters" on the horizontal axis), the percentage of drugs having at least one of their annotations enriched in their clusters over all annotated drugs (denoted by "Drugs" on the horizontal axis) and the percentage of Drug Categories enriched (denoted by "Categories" on the horizontal axis).

Cell line	Annotation type	Jaccard PPI	Jaccard COEX	Jaccard GI
A549	GO-BP	0.31	0.26	0.12
	RP	0.67	0.65	0.17
NHBE	GO-BP	0.29	0.31	0.08
	RP	0.69	0.6	0.35
CALU	GO-BP	0.31	0.23	0.11
	RP	0.69	0.62	0.08
Patient	GO-BP	0.17	0.19	0.08
	RP	0.56	0.56	0.13

Supplementary Table S1: **Enriched functions in infected and control networks are different.** For each cell line and patient data (column 1) we compute the Jaccard similarity between the enriched functions (GO-BP and RP terms, column 2) in the control and the infected iCells in the PPI network (column 3), in the COEX network (column 4) and in the GI network (column 5).

Cell line	Annotation type	#Uniquely enriched in infected
A549	GO-BP	872
	RP	202
NHBE	GO-BP	560
	RP	268
CALU	GO-BP	555
	RP	147
Patient	GO-BP	491
	RP	108

Supplementary Table S2: **Number of uniquely enriched functions in infected iCells.** For each cell line and patient data (column 1) we compute the number of uniquely enriched functions (GO-BP and RP, column 2) in the infected iCells (column 3).

Cell line	Network type	#Unique to infected	#Unique to control	#Common
A549	PPI	2,499 (1.39%)	1,399 (0.78%)	176,329 (97.84%)
	COEX	16,330 (2.69%)	14,393 (2.37%)	577,214 (94.95%)
	GI	254 (1.10%)	739 (3.19%)	22,164 (95.71%)
	iCell	375,321 (31.15%)	367,853 (30.53%)	**461,756 (38.32%)**
NHBE	PPI	1,707 (0.95%)	4,463 (2.49%)	173,185 (96.56%)
	COEX	12,965 (2.17%)	33,149 (5.54%)	552,212 (92.29%)
	GI	233 (1.01%)	1,069 (4.63%)	21,794 (94.36%)
	iCell	331,303 (28.72%)	365,157 (31.65%)	**457,217 (39.63%)**
CALU	PPI	9,683 (5.41%)	3,082 (1.72%)	166,147 (92.87%)
	COEX	60,724 (9.72%)	25,472 (4.08%)	538,825 (86.21%)
	GI	2,777 (13.12%)	663 (3.13%)	17,728 (83.75%)
	iCell	442,421 (37.25%)	382,304 (32.19%)	**362,863 (30.55%)**
Patient	PPI	2,313 (1.36%)	79,966 (46.87%)	88,318 (51.77%)
	COEX	24,112 (3.81%)	392,203 (61.92%)	217,101 (34.27%)
	GI	672 (3.23%)	11,837 (56.87%)	8,306 (39.90%)
	iCell	184,619 (18.62%)	671,946 (67.77%)	**134,930 (13.61%)**

Supplementary Table S3: **Numbers of unique and common edges between infected and control networks.** For the patient tissue and for each of the three cell lines (column 1) and for each molecular and iCell network (column 2), the table shows the number of unique edges in infected networks (column 3), the number of unique edges in control networks (column 4) and the number of edges common to infected and control networks (column 5).

Cell line	Network type	#Unique to infected	#Unique to control	#Common
A549	PPI	139 (1.43%)	108 (1.11%)	9,484 (97.46%)
	COEX	137 (1.46%)	104 (1.11%)	9,149 (97.43%)
	GI	87 (1.23%)	89 (1.26%)	6,881 (97.51%)
	iCell	139 (1.43%)	108 (1.11%)	9,484 (97.46%)
NHBE	PPI	107 (1.11%)	247 (2.56%)	9,284 (96.33%)
	COEX	104 (1.12%)	234 (2.51%)	8,970 (96.37%)
	GI	70 (0.98%)	232 (3.24%)	6,863 (95.79%)
	iCell	107 (1.11%)	247 (2.56%)	9,284 (96.33%)
CALU	PPI	482 (5.0%)	197 (2.05%)	8952 (92.95%)
	COEX	471 (4.96%)	191 (2.01%)	8830 (93.03%)
	GI	574 (8.07%)	153 (2.15%)	6383 (89.77%)
	iCell	482 (5.0%)	197 (2.05%)	8952 (92.95%)
Patient	PPI	269 (2.74%)	3905 (39.76%)	5647 (57.5%)
	COEX	264 (2.73%)	3839 (39.64%)	5581 (57.63%)
	GI	180 (2.6%)	3176 (45.9%)	3563 (51.5%)
	iCell	269 (2.74%)	3905 (39.76%)	5647 (57.5%)

Supplementary Table S4: **Numbers of unique and common nodes between infected and control networks.** For each cell line and patient data (column 1) and for each network type (column 2), the table shows the number of unique nodes in infected networks (column 3), the number of unique nodes control networks (column 4) and the number of common nodes in control and infected networks (column 5).

Cell line	A549	NHBE	CALU	Patient
A549	100	13	24	4
NHBE		100	18	4
CALU			100	11
Patient				100

Supplementary Table S5: **The gene overlap between the top 100 most rewired genes of infected and control iCells in different data.** For the patient lung sample (denoted by "Patient") and for each cell line (A549, NHBE, CALU), we compute the top 100 most rewired genes between infected and control iCells — the table shows the pairwise overlap between the top 100 most rewired genes among all the studied iCells.

Gene	Drug	Binding free energy (kcal/mol)	Dissociation constant (Molar)
ZNF35	NADH	-9.8	6.44×10-8M
RPSAP58	NADH		
ZNF562	NADH	-9.4	1.27×10-7M
OLFM2	Fostamatinib	-9.6	9.02×10-8M
CYB561	Zinc chloride		
ZNF41	Fostamatinib	-8.5	5.79×10-7M
LCMT2	N-Formylmethionine		
CSTF2T	NADH	-10.8	1.19×10-8M
NUP85	Cladribine	-7.2	5.21×10-6M
REEP4	Fostamatinib	-9.3	1.50×10-7M
ASRGL1	NADH	-9.7	7.62×10-8M
ZFP62	Artenimol	-7.6	2.65×10-6M
CBX5	Acetylsalicylic acid		
KLHL9	Artenimol	-10.6	1.67×10-8M
ZNF189	Fostamatinib	-9.9	5.43×10-8M
ZNF597	NADH	-10.8	1.19×10-8M
HIST2H2AC	Artenimol	-8.2	9.61×10-7M
CSTF1	Fostamatinib	-13	2.89×10-10M
ZNF507	NADH	-8.6	4.89×10-7M
ZNF286A	NADH	-10.7	1.41×10-8M

Supplementary Table S6: **Binding affinities of the predicted DTIs.** For each of the top 20 most rewired genes in patient iCells of infected versus control (column 1), we report the potential drug for repurposing based on our framework (column 2). Finally, for each predicted DTI we report its binding free energy (column 3, in kcal/mol) computed using AutoDock Vina v1.2 and its corresponding dissociation constant, K_d, (column 4, in Molar). Note that we could not find a experimentally validated, or predicted protein structure for the RPSAP58 gene, so we could not perform the docking for it. Also, we excluded from the drugs the small chemical compounds (zinc chloride, n-formylmethionine and acetylsalicylic acid).
